# Psychosis as a multisystem disorder of aberrant aging

**DOI:** 10.1038/s41514-026-00409-2

**Published:** 2026-05-29

**Authors:** Rachel Upthegrove, Amalie C. M. Couch, Antonio de Marvao, Benjamin I. Perry, Fabiana Corsi-Zuelli, Jack Rogers, Lahiru Handunnetthi, Nicholas M. Barnes, Nikolaos Koutsouleris, Paris A. Lalousis, Toby Pillinger, Zachary Freyberg

**Affiliations:** 1 Department of Psychiatry, University of Oxford, Oxford, UK; 2 Oxford Health NHS Foundation Trust, Oxford, UK; 3 Institute for Mental Health, University of Birmingham, Birmingham, UK; 4 Birmingham and Solihull Mental Health NHS Foundation Trust, Birmingham, UK; 5 Department of Women and Children’s Health, King’s College London, London, UK; 6 British Heart Foundation Centre of Research Excellence, School of Cardiovascular and Metabolic Medicine and Sciences, King’s College London, London, UK; 7 Medical Research Council Laboratory of Medical Sciences, Imperial College London, London, UK; 8 Centre for Human Brain Health, University of Birmingham, Birmingham, UK; 9 Centre for Human Genetics, University of Oxford, Oxford, UK; 10 Institute of Health Sciences, College of Medicine and Health, University of Birmingham, Birmingham, UK; 11 Max Planck Institute of Psychiatry, Munich, Germany; 12 Department of Psychiatry and Psychotherapy, Ludwig-Maximilian-University, Munich, Germany; 13 German Center for Mental Health (DZPG), partner site Munich-Augsburg, Munich, Germany; 14 Department of Psychosis Studies, Institute of Psychiatry, Psychology & Neuroscience, Kings College London, London, UK; 15 South London and Maudsley NHS Foundation Trust, London, UK; 16 Department of Psychiatry, University of Pittsburgh, Pittsburgh, PA USA; 17 Department of Cell Biology, University of Pittsburgh, Pittsburgh, PA USA

**Keywords:** Diseases, Neurology, Neuroscience

## Abstract

Psychotic disorders, including schizophrenia and affective psychosis, affect ~3% of the population and typically emerge in early adulthood. Cardiometabolic disease accounts for much of the 20-year life-expectancy gap in psychosis. Evidence indicates potentially causal processes, often seen in aging, act within and beyond the brain, and before the onset of treatment; these include inflammation, metabolic and mitochondrial dysfunction. Here we synthesize evidence and propose a framework that proposes psychosis as a multisystem disorder of accelerated aging, and outline implications for aging-targeted interventions.

## Introduction

Research on psychotic disorders, including schizophrenia (SCZ), has historically been dominated by a neurocentric perspective, centered on brain dysfunction. This includes previous evidence that cites schizophrenia as a disorder of neurodevelopment, with brain changes beginning in early life, or neurodegeneration, with degenerative processes interrogated through, for example, postmortem brain tissue. Whilst considerable evidence supports aberrant brain developmental processes^1^, and cognitive decline, with evidence of increased risk of dementias^2^, people with SCZ do not present with an inevitable course of progressive neurodegeneration. The focus on brain function, whilst a cornerstone of psychosis research, has recently been expanded to include the primary effect of illness visible in other organ systems. Cardiovascular and metabolic diseases are highly prevalent, with up to 50% increased prevalence for cardiometabolic disorders^3^. Crucially, elevated cardiometabolic risk coincides with illness onset or earlier and is present before the first exposure to antipsychotic drug treatment. This indicates that the heightened metabolic risk associated with psychosis cannot be explained solely by poor lifestyle or medication side effects^4,5^. First-episode psychosis cohorts consistently show elevated rates of insulin resistance, dyslipidaemia, and systemic inflammation prior to treatment^6–8^. These findings support reconceptualising psychosis as a multisystem disorder rather than one confined to the brain^9–14^. Moreover, signs of early biological aging appear across multiple domains in people with psychosis, with the strongest evidence from models of brain aging, but parallel signals in cardiovascular and metabolic systems.

The clinical consequences of these multisystem changes are profound for individuals with psychosis, who have a life expectancy 15–20 years shorter than the general population^15–18^, reflecting an accumulation of multimorbidity across the lifespan. Consistent with the hallmarks of aging, one-third of patients with first-episode psychosis exhibit chronic low-grade inflammation and oxidative stress, including raised circulating cytokines such as interleukin (IL)-6 and tumor necrosis factor (TNF)-α, and features of cellular immunosenescence^19,20^. Cardiovascular studies show arterial stiffness, subclinical atherosclerosis, and myocardial fibrosis^21,22^ in people with psychosis—abnormalities that cannot be fully explained by lifestyle or treatment exposure^4^.

Together, these findings support two central propositions: First, there may be a bidirectional interaction between brain and body in psychotic disorders. Second, SCZ should not solely be viewed through a brain health lens but as a condition with widespread effects, particularly in systems vulnerable to aging. Consequently, the high prevalence of cardiovascular, metabolic, and immune dysfunctions in individuals with psychotic disorders prompts a reconceptualisation of psychotic disorders from a neurocentric to a multisystem framework of aging^9–14^. Here, we position aging as a life-long process encapsulating the historic neurodevelopmental evidence and expanding this to include continuing aging-related mechanisms across organs and systems in psychotic disorders, including SCZ stages from first episode to treatment resistance. This rethinking of SCZ provides a novel foundation for examining psychotic disorders through the lens of aging biology, with implications for both mechanistic understanding and the development of interventions.

**Fig. 1:**
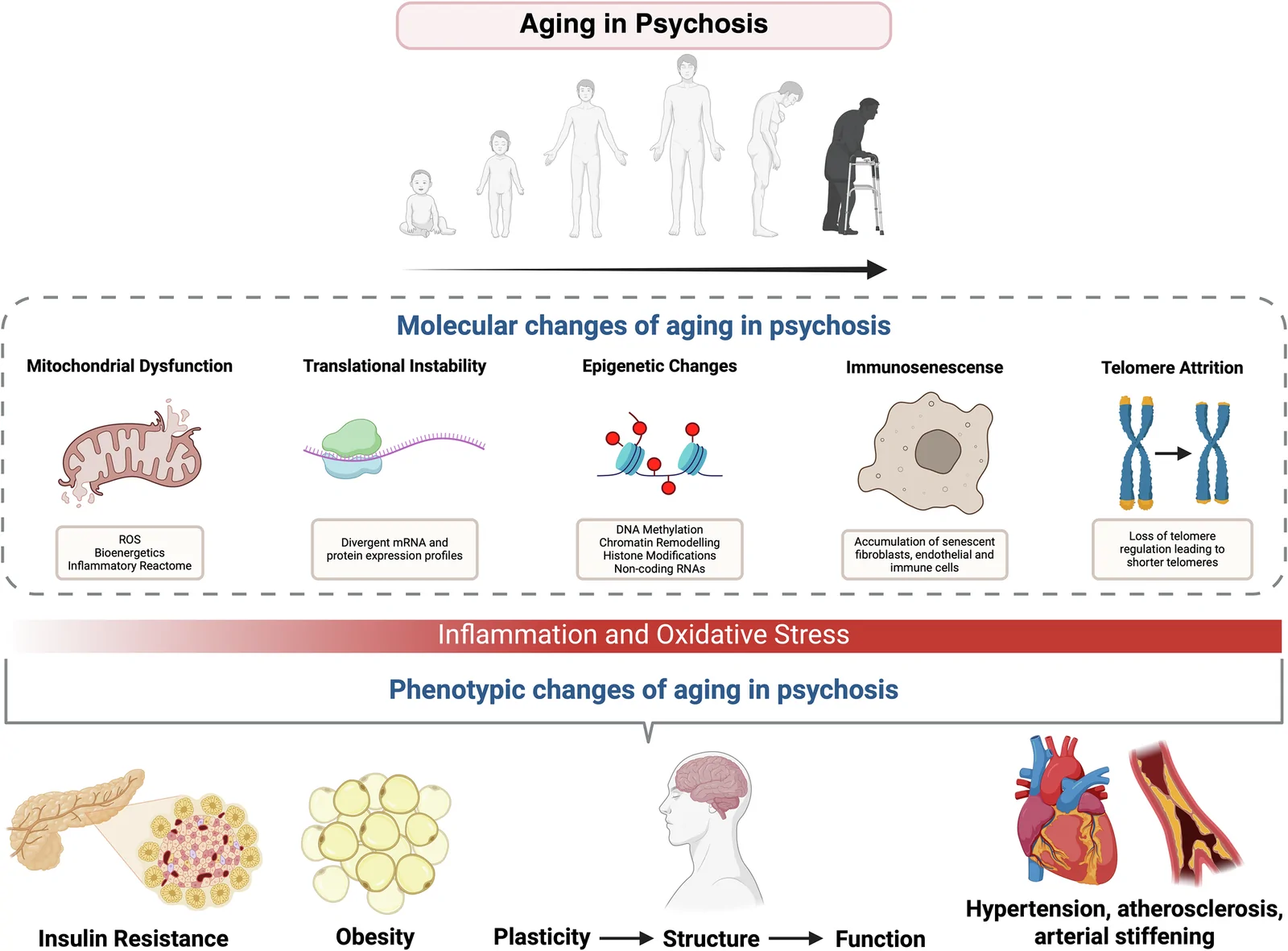
Molecular and phenotypic aging markers in psychotic disorders. including mitochondrial dysfunction, senescence, inflammation, and associated clinical outcomes (e.g., insulin resistance, neural decline). Based on the premise of key “Hallmarks of Aging” from Lopes-Otin et al.^206^, here we illustrate pathways and mechanisms currently under investigation in aging research that have relevance in psychosis: i.e., that also have some evidence of aberrance in psychosis models and data. Figure created in BioRender. Couch A. (2026).

This review aims to synthesize emerging evidence that psychotic disorders are characterized by accelerated biological aging across multiple systems in the brain and body. We propose an approach to psychosis that includes multisystem aging, underpinned by stress, chronic inflammation, and impaired metabolic regulation. To critically evaluate this hypothesis, we will integrate multidisciplinary findings from neuroimaging, proteomic, genomic, metabolic, and immunological studies, highlighting both supporting evidence and key knowledge gaps (Table 1). We refer to evidence across the stages of psychosis, and clarify where evidence is present in the first episode, in established SCZ, and in treatment resistance. We will also consider the therapeutic potential of this paradigm, including the development and repurposing of interventions targeting aging pathways, and the possibility of stratifying patients according to immuno-metabolic profiles (Fig. 1). By reframing psychosis as involving accelerated multisystem aging, our goal is to encourage integrative, whole-body approaches to research and treatment that may ultimately reduce psychosis-related multimorbidity and premature mortality.

**Table 1:** Evidence of multisystem advanced aging phenotypes in psychotic disorders, from both clinical and preclinical studies

Level	Domain	Age prediction model present?	Age prediction model applied to cohorts of psychotic disorders?	Evidence of aging in psychotic disorders	Citation
Organ	Brain	Yes	Yes	Gap between BrainAge and chronological age increased (~5 years) in individuals with psychotic disorders; associated with cortical thinning, gray matter loss, oxidative stress, and inflammation.	^27–36,212^
	Proteomic /Inflammatory	Yes	Indirectly	Key proteins driving ProteomicAge prediction models (e.g., GDF15, CXCL9) are elevated in psychotic disorders; overlap between aging and psychotic disorder inflammatory profiles.	^25,42,48–50,57^
	Cardiovascular	Yes	No	ECG and CMR models of cardiac aging not yet applied in psychotic disorders, but individuals with psychotic disorders show arterial stiffness and myocardial fibrosis independent of lifestyle.	^4,13,22,62,64,213,214^
	Metabolic	Yes	No	No metabolomic age models yet used in psychotic disorders; however, early insulin resistance and shared genetic risk between psychotic disorders and T2DM suggest premature metabolic aging.	^6,65–72^
Molecular	Mitochondrial dysfunction	No	No	Mitochondrial alterations observed in psychotic disorders include reduced oxidative phosphorylation, increased ROS, and altered ultrastructure; link to cellular aging and energy imbalance.	^74–78,88,215^
	Cellular and immunosenescence	No	No	Elevated SASP markers (e.g., IL-6, CXCL9), T-cell aging, and glial senescence observed in psychotic disorders; overlap with inflammaging and senescent phenotypes.	^109,122,124,126,127^
	Genomic and epigenomic instability	Yes	Yes	Accelerated epigenetic aging found in SCZ cohorts; shorter telomeres, reduced telomerase activity, and damaged mitochondrial DNA also observed.	^143–153^
	Translational regulation	No	No	Disruption in mRNA-protein correlation in SCZ; loss of compensatory translation may underlie early neurotransmitter dysfunction and contribute to brain aging.	^154–158^

## Evidence of aberrant aging in psychosis

The ability to quantify aging processes has been transformed by the development of epigenetic clocks, which first estimated biological age based on DNA methylation patterns^23,24^. Advances in machine learning have enabled the emergence of multimodal aging clocks, including proteomic^25^ and neuroimaging-based models^26^, that draw on large-scale datasets to quantify an individual’s biological age. These models can calculate an “age gap”: the difference between predicted biological age and chronological age, providing a measurable index of accelerated or decelerated aging in specific tissues or systems. The age gap can therefore quantify differences in biological aging between healthy individuals and those with illnesses, including SCZ.

### Brain aging

The concept of “BrainAge” refers to an estimate of biological brain age derived from neuroimaging data, most often structural magnetic resonance imaging. Early approaches used linear regression models relating imaging features such as cortical thickness or gray matter volume to chronological age. More recent studies apply machine learning methods that can capture complex, nonlinear relationships across thousands of voxels. In both cases, the difference between predicted and actual age, termed the “brain age gap” (BAG), provides a quantitative index of accelerated or decelerated brain aging. BrainAge models are the most consistently applied method to quantify increased aging in psychotic disorders. Longitudinal^27–29^ and cross-sectional^30,31^ MRI studies in SCZ consistently demonstrate that the brain appears approximately 5 years older than expected for chronological age^32^. This BAG is associated with reduced gray matter volume, particularly in the orbitofrontal and limbic regions, and with cortical thinning^33–36^, and is evident even in early stages of illness, as highlighted by the ENIGMA-Schizophrenia consortium^34^. It remains unclear whether the BAG reflects processes that begin before psychosis onset, driven in neurodevelopment largely via genetic factors, or is instead shaped by illness progression, medication effects, or lifestyle factors^37,38^. Further longitudinal imaging studies spanning the period before psychosis onset are needed to address this key knowledge gap. Risk for dementia is increased in people with SCZ^2^, and this also highlights our proposal of a life course view aging processes, and this also highlights our proposal of a life course view aging process.

Mechanistic clues for the BAG come from data demonstrating that structural changes associated with BrainAge may be linked to active processes such as oxidative stress, glutamatergic dysfunction, and bioenergetic impairment^32,33,35^. Meta-analyses also show that individuals with psychosis exhibit low-grade systemic inflammation prior to illness onset^8,10^, but levels of centrally-measured glutathione—the brain’s primary antioxidant—are initially preserved and then decline as the illness progresses^32,39^. This antioxidant depletion, together with sustained inflammation, may drive a shift toward glycolytic metabolism in the brain at the expense of efficient mitochondrial ATP production^32^. Thus, BrainAge is not merely a predetermined biomarker of disease severity but reflects downstream consequences of inflammation and cellular stress, reinforcing the potential for interventions as a brain–body condition with aging-relevant pathology.

### Proteomic and inflammatory aging

Proteins are central effectors of all biological aging processes. Evidence suggests that chronic inflammation emerges from the loss of proteostasis: the dynamic balance between protein synthesis and degradation^40^. This loss impairs the cell’s ability to maintain a stable, functional proteomic environment, resulting in biological aging^41^. Several blood-based aging models have emerged, referred to as proteomic or inflammatory aging clocks, which represent a novel approach to quantifying biological age from circulating protein signatures^25,42,43^. These models, often derived from broad-spectrum panels, accurately predict chronological age and allow estimation of an individual’s proteomic or inflammatory age gap: the difference between predicted biological and actual age. A larger age gap is consistently associated with increased risk of multimorbidity, mortality, and age-related diseases such as stroke, type 2 diabetes mellitus (T2DM), and neurodegeneration^25,42,43^.

Even within the broad proteomic models, inflammatory proteins dominate the top contributors to biological age, highlighting the centrality of immune signaling in aging. One UK Biobank (UKBB)-derived model using ~3000 proteins identified CXCL17 and GDF15 amongst others as part of the top 20 protein predictors of accelerated proteomic aging^25^. Recent studies further defined organ-specific proteomic aging models from the UKBB dataset, demonstrating that brain-specific proteomic aging contains neuroinflammation as a key enriched pathway involving proteins such as matrix metalloproteinase-9 (MMP-9), the pro-inflammatory TNFα receptor, TNFRSF1B (TNFR2), and the myeloid cell receptor for adhesion, ITGAM (CD11B)^43^. The involvement of these proteins in biological processes such as inflammatory signaling, glial reactivity, and extracellular matrix remodeling suggests a mechanistic link through which systemic inflammation contributes to brain circuit vulnerability in aging.

These findings are consistent with independent inflammatory aging models. Such models highlight CXCL9, a chemokine that attracts T-cells to sites of inflammation^44^, as a strong contributor involved in cardiac aging, adverse cardiac remodeling, and poor vascular function^42^. MMP-9, a member of the matrix metalloproteinase family of zinc-dependent extracellular and membrane-bound endopeptidases that cleave components of the extracellular matrix, is implicated in synaptic remodeling and neuroplasticity^45^, activity-dependent processes critical for memory and cognitive function^46^. Since appropriate MMP-9 activity is necessary for healthy brain development and function^47^, dysregulated expression may disrupt synaptic integrity and impair plasticity, particularly in aging or chronic inflammation. Aging-associated increases in MMP-9 may therefore alter brain function and plasticity both directly, by degrading structural components required for synaptic support, and indirectly, through interactions with neuroinflammatory signaling cascades. Indeed, in the general population, individuals with youthful brain and immune proteomic profiles had lower levels of inflammatory proteins like MMP-9 and higher levels of neuroprotective extracellular matrix components such as BCAN and NCAN^43^. These data suggest that preservation of brain extracellular matrix and suppression of chronic inflammation are central to healthy aging.

Importantly, many of the blood-based markers that drive proteomic aging models are elevated in individuals with psychotic disorders, suggesting a shared phenotype between psychotic disorders and biological aging. For example, GDF15 - a cytokine induced by mitochondrial stress and a core marker of age-related disease burden - is elevated in psychotic disorders^48,49^. CXCL9 acts as an important link between aging, immune dysregulation, and multimorbidity^42,50^ and is elevated in SCZ^51^. Furthermore, two of the most weighted features driving Oh et al.’s brain-specific proteomic age model^43^ were (1) upregulation of neurofilament light chain (an important marker for several neurodegenerative diseases^52^), and (2) downregulation of complement proteins such as C1QL2^53^, which plays a role in synaptic stabilization. Both are raised in the plasma of individuals with psychotic disorders compared with controls^54,55^. In the same model, individuals with an older proteomic brain age exhibited increased levels of MMP-9^43^, whose activity is increased in the early-phase of psychotic disorders^56^. A recent gene ontology analysis showed an 18-fold association of proteomic age gap with the term “modulation of excitatory postsynaptic potential”^57^. This suggests that aging-related proteomic changes affect neuronal signaling and synaptic plasticity as well as immune dysfunction.

Levels of peripherally measured inflammatory proteins do not match the levels seen in major inflammatory conditions such as rheumatoid arthritis or infection. However, even modestly raised cytokine levels show association with behavioral and peripheral impact^58,59^. and evidence from these genomic studies and our own Mendelian randomization suggest a potential causal relationship between low-level chronic inflammation and brain structure, and other organ impact^60^. Inflammatory and proteomic signals may also be downstream of autonomic dysfunction^61^, which has considerable evidence in mental illness and aging literature. Together, these findings indicate that proteomic and inflammatory signatures of aging may be detectable at or before the onset in late adolescence/early adulthood and overlap with evidence of age-related multimorbidity in the older general population.

### Cardiovascular aging

Cardiovascular aging is characterized by progressive structural and functional deterioration of the heart and vasculature, including myocardial remodeling, diastolic impairment, myocardial fibrosis, arterial stiffening, and endothelial dysfunction^62^. These processes are partly driven by chronic inflammation. Because distinct cohorts of patients with psychotic disorders are themselves characterized by persistent low-grade inflammation, affected individuals may be particularly vulnerable to accelerated cardiovascular aging, contributing to their markedly elevated burden of cardiovascular morbidity and premature mortality.

Converging evidence indicates that individuals with psychotic disorders show early cardiovascular pathology consistent with accelerated aging. Increased arterial stiffness, most often assessed by aortic pulse-wave velocity and regarded as a hallmark of vascular aging, is evident in individuals with SCZ, including at first-episode psychosis and in unmedicated cohorts, suggesting intrinsic vascular dysfunction^13^.

Cardiac MRI studies show concentric left ventricular remodeling and parametric-mapping changes compatible with diffuse fibrosis in psychosis compared to healthy controls matched for age, sex, ethnicity, smoking status, and BMI, indicating that these alterations cannot be fully explained by lifestyle or treatment effects^4,22^. Genetic data strengthened this picture: higher polygenic risk for schizophrenia associates with smaller cardiac volumes and reduced diastolic function, implicating immune and pro-fibrotic TGF-β pathways and pointing to intrinsic myocardial stiffening^4,22,63^.

Beyond observational studies, recent work has developed quantitative models of cardiovascular aging. Baek et al. developed a deep learning model to predict cardiac age from 12-lead electrocardiograms (ECGs)^64^, showing that a 6-year gap between predicted and chronological cardiac age strongly predicted all-cause mortality and major adverse cardiovascular events^64^. Building on this, Shah et al. used multimodal cardiovascular imaging and machine learning in nearly 40,000 UKBB participants to derive a comprehensive cardiovascular age metric^62^. Accelerated cardiovascular aging in this model was associated with cardiometabolic risk factors, multimorbidity, and reduced health span, and was enriched for genetic variants involved in sarcomere integrity, immune regulation, and stress-response pathways. Although these models have not yet been applied in SCZ, their mechanistic insights are highly relevant. They suggest that the vascular stiffness, myocardial fibrosis, and impaired elasticity in psychotic disorders may reflect convergence on shared biological aging pathways. Applying cardiovascular aging models to psychosis cohorts could provide scalable biomarkers of psychosis-related cardiovascular risk and identify potential therapeutic targets.

### Metabolic and metabolomic aging

Metabolic aging encompasses gradual impairments in glucose homeostasis, lipid metabolism, and mitochondrial function, contributing to age-related disorders such as T2DM and cardiovascular disease. In psychotic disorders, metabolic dysfunction is prevalent, even at early stages. Approximately one-third of individuals with first-episode psychosis show evidence of insulin resistance in the absence of antipsychotic drug exposure^6^. Several of the mechanisms associated with metabolic dysfunction, particularly dysregulation of key inflammatory pathways, are shared with the biology of aging, supporting the hypothesis that psychotic disorders involve premature metabolic senescence. Importantly, genomic studies show shared risk architecture between psychotic disorders and T2DM, with several loci related to insulin signaling, lipid metabolism, and inflammation implicated in both conditions^65^. These findings support the notion that psychotic disorders are not only comorbid with, but mechanistically intertwined with, accelerated metabolic aging.

Metabolomic aging clocks extend this framework by predicting biological age based on the profile of circulating metabolites^66–68^. The MileAge model, estimating age using 128 plasma metabolites from UKBB, found individuals with a high MileAge were frailer, had shorter telomeres, and had higher all-cause mortality^66^. Faquih et al. predicted metabolomic age from 826 metabolites in healthy donor blood from the INTERVAL study. They found that BMI and obesity were associated with an increased metabolic age gap, while no direct associations were identified for T2DM, hypertension, depression, or sleep duration^68^. Finally, the generation of a metabolic aging score from 325 nuclear magnetic resonance biomarkers in UKBB by Zhang et al. predicted the onset of 14 diseases, most notably T2DM and hypertension^67^. These clocks capture subtle, system-wide metabolic changes that correlate with morbidity and mortality to offer a tool for quantifying metabolic age. Yet, no such models have been applied to cohorts of people with psychotic disorders. Nevertheless, psychiatry-specific tools demonstrate the feasibility of tailored metabolic risk prediction. The PsyMetRiC tool, for example, is a metabolic risk calculator developed to estimate the likelihood of developing metabolic syndrome specifically in people with psychotic disorders, with extensive external validation and evidence of accuracy across multiple studies^69–72^. While it is not a biological aging clock, PsyMetRiC incorporates age as a critical risk factor with appropriate calibration for psychotic disorders, where risk begins in early adulthood, therefore giving accurate individualized risk estimates. PsyMetRiC may therefore serve as a pragmatic bridge between metabolic risk and aging research in psychotic disorders, especially if integrated with Brain, Proteomic, and Cardiac Age predictions.

Overall, convergent evidence across multiple biological domains points to SCZ as a disorder of multisystem accelerated aging. Applying and integrating these aging models holds promise for advancing mechanistic understanding, identifying biomarkers for risk stratification, and informing novel treatments to reduce multimorbidity and premature mortality.

## Cellular and molecular mechanisms

### Mitochondrial dysfunction in psychosis

**Fig. 2:**
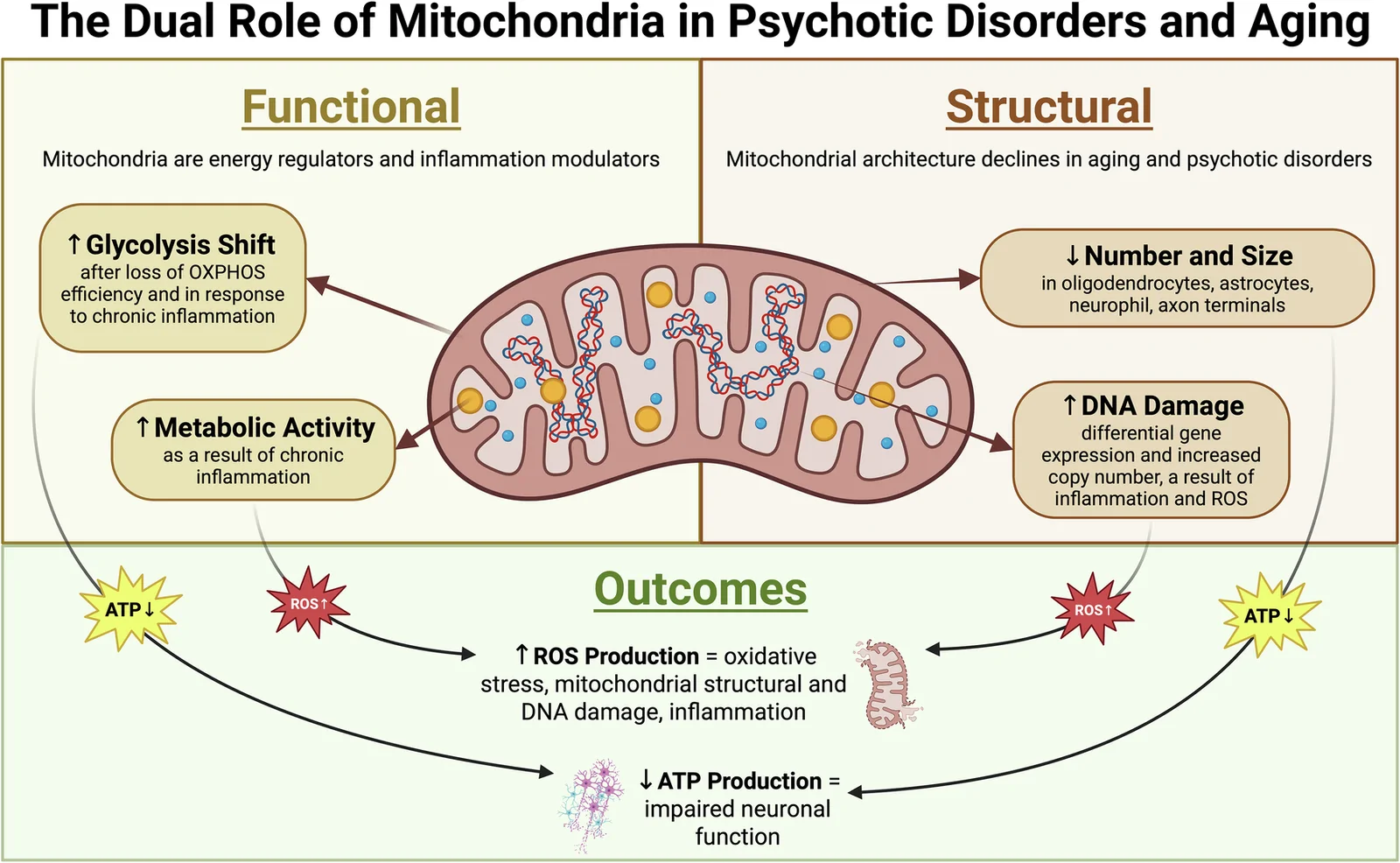
The dual role of mitochondria in psychotic disorders and aging. Functionally, mitochondria act as energy regulators and inflammation modulators. In psychotic disorders, chronic low-grade inflammation leads to a shift from oxidative phosphorylation (OXPHOS) to glycolysis and an increase in mitochondrial metabolic activity, both contributing to elevated reactive oxygen species (ROS) production. Structurally, aging and psychosis are associated with reduced mitochondrial number and size in key brain cell types (e.g., oligodendrocytes, astrocytes, neuronal axon terminals), as well as increased mitochondrial DNA damage and altered gene expression. These functional and structural changes converge to increase ROS and decrease ATP production, resulting in impaired neuronal function and contributing to both central (e.g., cognitive deficits) and peripheral (e.g., metabolic comorbidities) manifestations of psychotic disorders. Figure created in BioRender. Couch A. (2026).

Advances in treatments for psychotic disorders that target accelerated biological aging are limited by the lack of specific mechanisms, particularly at the cellular level, in relevant patient populations. One potential target is mitochondrial dysfunction, which has been documented across multiple brain regions in postmortem studies of individuals with SCZ^73^, both driven by and contributing to chronic low-grade inflammation^41^ (Fig. 2).

Balanced energy metabolism is an essential prerequisite for brain health. The brain lacks extensive energy storage capacity, relying on the supply of glucose from blood, making it particularly vulnerable to metabolic dysfunction. Shifts from oxidative phosphorylation (OXPHOS) to glycolysis have been observed both peripherally and centrally in psychotic disorders^74–76^, and this may contribute to neuroinflammation and oxidative stress (Fig. 2). These same processes underlie cardiometabolic comorbidity, further supporting potential shared mechanisms across psychotic disorders rooted in disturbed energy balance. Mitochondria are central to energy metabolism, producing ATP via OXPHOS, but are also key generators and targets of reactive oxygen species (ROS). Elevated ROS levels, impaired OXPHOS, and mitochondrial damage are common in aging tissues and are also evident in psychotic disorders^77,78^. Moreover, preclinical data in the Drosophila model show that within dopamine neurons, elevated mitochondrial ROS levels are sexually dimorphic, with males more affected than females^79^. This raises the possibility that sex differences in mitochondrial ROS drive males’ greater vulnerability to FEP compared to females.

Reduced mitochondrial number and size are documented in oligodendrocytes^80^, astrocytes^81^, neuropil^82^. and axon terminals^82^ in the anterior cingulate cortex^83^, prefrontal cortex^84^, frontal cortex white^85^ and gray^80^ matter, hippocampus^81^, caudate^86^, and caudate putamen^82^ cortical differences in SCZ postmortem tissue (Fig. 2)^87^. Support for mitochondrial function being a primary driver, rather than secondary to environmental effects, exists: in antipsychotic drug-naïve individuals and at clinical high-risk for psychosis, mitochondrial complex III function and lactate levels correlate with deficits in motivation symptoms^88^. Complex I dysfunction has also been observed in SCZ^89,90^. However, multiple studies have shown that antipsychotics can disrupt mitochondrial function by inhibiting respiratory chain complexes^91–93^, depolarizing mitochondrial membranes^94–96^, altering gene expression in energy metabolism pathways^97–101^, and promoting mitochondrial dysfunction linked to metabolic side effects^99,102^.

Human induced pluripotent stem cell (hiPSC) models enable real-time interrogation of mitochondrial biology directly in patient-derived cells. These cells are reprogrammed to maintain the patient’s genotype while operating in an experimental environment free from the confounding influence of antipsychotics. Studies using hiPSC-derived cortical neurons from individuals with psychotic disorders noted a differential transcriptional response to Interferon (IFN)-y compared with healthy controls, with mitochondrial *NDUFA2/3* gene expression showing the most divergent response^103^.

Together, functional changes in mitochondrial biology associated with psychotic disorders are indicated by diminished respiratory complex activity and accumulation of cytotoxic ROS that result in changes in neuronal physiology. Structurally, such mitochondrial alterations are reflected by disruptions in mitochondrial ultrastructure^80–83,85,86^ and in the function of respiratory complexes^88–90^ in individuals with psychotic disorders. These data suggest both peripheral and central mitochondrial vulnerability is associated with psychotic disorders, which may contribute to neural circuit dysfunction and somatic comorbidities such as T2DM and cardiovascular disease—conditions highly prevalent in psychotic disorders and accelerated by mitochondrial aging^104^. We therefore propose that cellular stress may be driving chronic inflammation and accelerated aging, and this is reflected in impaired mitochondrial structure and function (Fig. 2).

### Cellular senescence and immunosenescence in psychosis

Cellular senescence is a key hallmark of aging^41^, defined by stable cell cycle arrest accompanied by the pro-inflammatory senescence-associated secretory phenotype (SASP)^105–107^. In SASP, senescent cells secrete cytokines, chemokines, and proteases that contribute to tissue dysfunction and chronic inflammation. Typically, immune cells help clear senescent cells, but with aging, these immune mechanisms degrade—a process termed immunosenescence^108^. Together, this generates a self-reinforcing loop that contributes to the accumulation of senescent cells and “inflammaging,” the chronic low-grade inflammation present during biological aging^109,110^. Such accumulation evokes the detrimental effects of SASP proteins on the extracellular matrix and progenitor cell function^111^, which then drives age-related musculoskeletal diseases^112^ (e.g., osteoporosis^113^, osteoarthritis^114^, sarcopenia^115^), as well as pulmonary fibrosis^116^, T2DM^117^, and dementia^118–120^. There is emerging evidence suggesting that cellular senescence and immunosenescence may also be active in psychotic disorders.

First, recent work shows elevated SASP indices (a quantified score based on several SASP proteins)^121^ in individuals with severe mental illness. In patients with BD, increased expression of SASP components has been reported^122^, while MRI studies in late-life depression show correlations between SASP expression, age, and mean diffusivity of the left and right cingulate bundles^123^. Though composite SASP indices remain unstudied in SCZ compared to BD, there is strong evidence in SCZ cohorts that individual cytokines within the SASP profile, such as IL-6, TNF-α, and CXCL9, correlate with psychotic disorders and particularly those subgroups with poor outcomes^109,124^. Psychotic disorders may therefore represent a state of premature inflammaging where senescent signaling and immune dysfunction converge. Supporting this, a mouse model of accelerated senescence shows shrinkage, loss, and retraction of cortical neurons and their dendrites, loss of dendritic spines and synapses, as well as impaired learning and memory^125^. This suggests that senescent signaling can drive both brain structural and functional deficits in key areas relevant to psychotic disorders.

Second, T-cell senescence may contribute to the immune dysfunction observed in psychotic disorders^126,127^. Aging impairs T-cell activation and function, particularly affecting CD4⁺ T cells and regulatory T cells (Tregs)^128^, the latter playing a key role in maintaining immune homeostasis, suppression of inflammation, and prevention of autoimmunity^129,130^. SCZ-associated genetic variants are enriched in regulatory regions of activated CD4⁺ T cells^131^, and experimental models show that Tregs support early brain development by modulating glial activity^129,132^. Dysfunctional Treg signaling, especially involving disinhibition of the IL-6/STAT3 pathway, can lead to glial activation, impaired microglia–T cell communication, and disrupted synaptic pruning, potentially underlying cognitive deficits in psychotic disorders^133^. Tregs are especially vulnerable to aging, with aged Tregs losing their suppressive and neuroprotective functions, including their ability to support remyelination and prevention of neuroinflammation^134^. In rodents, expansion of brain Tregs can reverse neuroinflammation and cognitive decline in aging models^135^.

Immune profiling in psychotic disorders has been limited to basic blood markers such as cytokines, total lymphocytes, and monocytes^136^, constraining insight into mechanistic immunopathology. However, the few flow cytometry studies available suggest that lower Treg frequencies are associated with greater cognitive and negative symptom severity in psychotic disorders^130^. These require replication in larger cohorts of minimally treated early-stage psychosis individuals, as well as quantifying the potential impact of these cellular changes upon the functional immunosuppressive activity. Deeper immunophenotyping with single-cell analyses and functional evaluation may clarify how aging-related immune decline intersects with the pathophysiology of psychotic disorders.

Recent work highlights that microglial senescence is a key driver of brain aging. In both aging and Alzheimer’s disease models, microglia adopt a senescent phenotype characterized by persistent secretion of senescence-associated cytokines and maladaptive synaptic pruning^137^. Importantly, these are not simply hyperactive cells but represent a distinct population with stable senescent signatures. For example, recent studies identified TREM2-expressing senescent microglia in ageing models and demonstrated that their selective elimination with senolytic therapy reduced neuroinflammation and improved cognitive performance^138^. These mechanistic insights align with observations in SCZ, where microglial abnormalities, including excessive complement-mediated pruning^139^ and chronic pro-inflammatory activation^140,141^ have been repeatedly documented. Together, these findings raise the possibility that SCZ involves the premature emergence of senescent-like microglial states, contributing to immune aging. Functional study of human brain isolated microglia is clearly challenging, but the opportunity to differentiate peripheral blood monocytes into microglia that likely maintain epigenetic as well as genetic signatures allows direct investigation of these states in cells from patients in comparison to controls. Such studies, along with studies applying single-cell transcriptomics, validated senescence biomarkers, and in vivo imaging in psychosis cohorts, will be essential to establish whether microglial senescence is a defining feature of the disorder and a potential therapeutic target.

Overall, evidence suggests that the aging hallmarks of cellular senescence and immunosenescence could be active, measurable, and clinically relevant processes in psychotic disorders.

### Genomic, epigenomic, and translational instability in psychosis

With increased aging comes progressive genomic instability, including point mutations, chromosomal rearrangements, telomere shortening, and both nuclear and mitochondrial DNA (mtDNA) damage^41^. These changes result from cumulative exposure to oxidative stress, replication errors, and declining DNA repair efficiency with age^142^. In psychotic disorders, similar patterns of genomic disruption may reflect or contribute to accelerated aging. Notably, mtDNA damage may be altered in active psychosis. Unmedicated, first episode actively psychotic individuals show increased mtDNA copy number versus medicated individuals, and they have elevated levels of oxidative DNA damage (8-oxodG) compared to healthy controls^143^. When corrected for platelet-to-leukocyte ratio, whole blood mtDNA copy number also decreased with age, illness severity, and cumulative exposure to antipsychotics^144^. These effects were replicated in vitro, where therapeutic doses of antipsychotic drugs reduced mtDNA copy number in stem cell–derived neurons^144^. Such findings demonstrate the impacts of mtDNA instability on mitochondrial dysfunction (see the section “Mitochondrial dysfunction in psychosis”), aging, and psychotic disorders.

Telomere attrition, another marker of genomic aging^41^, is also accelerated in psychotic disorders^145–148^. Individuals with SCZ, particularly those with treatment resistance, show shorter leukocyte telomere length^146,148^ and impaired telomerase activity^145^. Lower expression of telomerase reverse transcriptase (TERT), along with greater TERT/telomere length ratios, indicates compromised telomere maintenance in individuals with early-stage psychotic disorders [age of psychotic individuals (29 ± 9 years); controls (31 ± 8 years)]^145^. These alterations mirror changes in aging-related diseases such as pulmonary and kidney fibrosis^149,150^.

Aging and psychotic disorders also share a common feature of epigenetic dysregulation^41,151^, including altered DNA methylation, histone modifications, and chromatin remodeling. Accelerated epigenetic age, as measured by DNA methylation clocks, is associated with symptom severity, metabolic risk, and early-life adversity in psychotic disorders^152^. Postmortem studies have identified widespread methylation differences in the frontal cortex, including at loci involved in glutamatergic and GABAergic signaling, brain development, and immune regulation^153^. A systematic review by Smigielski et al. further highlighted consistent alterations in DNA methylation and microRNA (miRNA) expression across patient cohorts, though heterogeneity and cross-tissue extrapolation remain challenges^151^.

Impaired translational regulation, which disrupts protein synthesis from mRNA, may also contribute to accelerated aging in psychotic disorders. Translational dysregulation contributes to another hallmark of aging: loss of proteostasis, which is the delicate balance of protein production, folding, and degradation essential for cellular health^41,154^. Divergent mRNA and protein expression profiles have been observed in psychotic disorders, particularly in brain regions like the dorsolateral prefrontal cortex^155–158^. If translational regulation is disrupted in psychotic disorders, leading to proteostatic loss, this could result in early neurotransmitter deficits and cellular stress, even in the absence of overt cell loss^154^. In addition, microRNA (e.g., miR 137 and miR 34)^159^ changes are increasingly examined in neurodevelopmental models and early onset SCZ, and add to the evidence of progressive illness course. Collectively, these findings suggest that psychotic disorders are associated with disruptions in genomic, epigenetic, and translational instability—all hallmarks of aging.

## Clinical implications, future directions, and conclusions

### Translational models

Although postmortem studies have identified aging-related pathology in psychotic disorders, they are limited by the lag between illness onset and tissue sampling, as well as by the scarcity of samples spanning critical developmental windows^160^. Patient-derived in vitro cellular models provide a powerful alternative for probing mechanisms of accelerated aging during these vulnerable periods^160^.

One promising approach uses transdifferentiated cells that retain age- and disease-related epigenetic signatures, such as induced monocyte-derived microglia (iMDMs)^161^ and induced neurons (iNs)^162,163^. For instance, iMDMs from individuals with SCZ exhibited increased synaptic engulfment, which was ameliorated in vitro by minocycline treatment^164^, pointing to an immune-mediated mechanism with therapeutic potential. Despite this, minocycline was ineffective in two large SCZ trials, perhaps highlighting the importance of stratifying patients by underlying biology when evaluating targeted interventions^165,166^. The ability to generate iMDMs and microglia from iPSCs from the same patient’s cells offers an opportunity to investigate epigenetic versus genetic factors responsible for modifying aging processes.

A complementary strategy involves hiPSC-derived models^167^ to investigate inflammation, mitochondrial dysfunction, and immune–metabolic interactions^160^. hiPSC-derived astrocytes from individuals with SCZ display lower baseline levels of NLRP3 inflammasome components, heightened caspase-1 activity after stimulation, and impaired glycolytic responses to suggest intrinsic bioenergetic vulnerability^168^. Similarly, hiPSC-derived cortical interneurons (cINs) from individuals with SCZ co-cultured with activated microglia show reduced arborization, disrupted metabolic pathways, and impaired GABAergic synapse formation^169^. However, a limitation of hiPSC models is that they typically resemble fetal or early developmental cell states^160^, which may not capture aging phenotypes unless exposed to artificial aging protocols, such as GENtoniK^170^.

Taken together, these in vitro models offer critical insight into the cellular and molecular underpinnings of accelerated aging in psychotic disorders, while also enabling high-throughput screening of potential interventions and preclinical validation of novel drug targets.

### Biomarker and novel intervention targets

Validated novel, non-invasive aging biomarkers such as BrainAge and ProteomicAge provide new avenues for stratifying individuals with psychotic disorders into high-risk groups prior to clinical trials. Such an approach aligns with precision psychiatry to improve treatment targeting. Current experimental medicine studies involving first episode patients, such as OPTiMiSE^171^ and PIMS^172^, already demonstrate the feasibility of stratifying patients for better trial outcomes, either by symptoms assessed using the Positive and Negative Syndrome Scale scale or by plasma IL-6, respectively. Incorporating biological aging indices into future intervention trials will be critical for identifying those most likely to benefit from immune–metabolic therapies or early prevention strategies.

Evidence from experimental models and pilot clinical trials supports the potential of anti-inflammatory agents, such as IL-6 inhibitors^172^, to mitigate low-grade chronic inflammation associated with accelerated aging in psychotic disorders. Several small trials have investigated targeted immunotherapies in psychotic disorders. Agents like tocilizumab (anti-IL-6R)^172–174^, canakinumab (anti-IL-1β)^175^, and adalimumab (anti-TNF-α)^176^ have shown mixed results, with some improving specific symptom domains such as cognition or negative symptoms, particularly in patients with elevated baseline inflammation. Other immune-modulating agents, such as rituximab (anti-CD20)^177^, fingolimod (S1P modulator)^178^, and methotrexate (Treg-restoring immunosuppressant)^178^, have also shown promise in small studies, improving global functioning or specific symptom domains. Collectively, these early human trials suggest that targeting immune dysregulation, a core feature of accelerated aging in psychotic disorders, could yield therapeutic benefits, particularly when stratification by inflammatory status guides treatment allocation. This reflects a broader shift toward repurposing immuno-metabolic therapies and underscores the importance of early intervention in psychotic disorders, particularly during the prodromal or first-episode phase.

In addition to anti-inflammatory agents, several other emerging interventions could target biological aging mechanisms in psychotic disorders. First, senolytics, a class of compounds designed to selectively eliminate senescent cells^179^, can reduce systemic inflammation and improve tissue function in age-related disease in rodents^180–182^, making senolytics a compelling therapeutic avenue in psychotic disorders where cellular senescence is elevated^183^. Second, N-acetylcysteine (NAC), an antioxidant and glutathione precursor, has shown promise in targeting age- and psychosis-related oxidative stress and mitochondrial dysfunction^184^. NAC has already demonstrated benefits on negative symptoms and cognition in clinical trials^185^, and its role in redox regulation may make it especially relevant for slowing cellular aging. Third, psychedelics and PIPIs (Psychedelic drug Informed but Psychedelic experience Inactive)^186^ have been shown to reduce inflammation^187,188^ and particularly neuroinflammation^189–191^. Recent work further suggests that psilocybin and psilocin may influence multiple hallmarks of aging, such as extending lifespan in mice, delaying cellular senescence, lowering ROS production, preserving telomere length, and modulating the expression of proteins implicated in longevity, such as sirtuin-1^192^.

Lastly, metabolic therapeutics such as metformin have demonstrated positive outcomes in managing metabolic dysfunction in an FEP cohort, as assessed by weight management^193–195^. Beyond weight management, metformin may also target core pathophysiological processes relevant to psychosis and aging simultaneously, including insulin resistance, low-grade inflammation, and impaired mitochondrial bioenergetics^196,197^. It is important to measure metabolic impacts themselves in the periphery, e.g., cardiometabolic outcomes; however, there is also growing evidence that peripherally acting agents may have downstream effects in brain function, including low-dose interleukin 2, which expands T-regulatory cells in the periphery, that can reduce central inflammation and may improve symptoms of depression^198^. Thus, the question is whether treatments need to be brain penetrant. Similarly, glucagon-like peptide-1 receptor agonists (GLP-1RAs), such as semaglutide, are being evaluated in clinical trials for their potential to improve metabolic health^199^. In addition to metabolic benefits, GLP-1 receptor activation exerts anti-inflammatory^200,201^, antioxidant^202^, cardioprotective^202,203^, and neuroprotective^204,205^ effects, supporting its relevance for this patient population^199^. Consistent with the rationale that psychotic disorders may represent metabolically-driven aging diseases, a large cohort study by Xie and colleagues using US Veterans Affairs data found that GLP-1RA use was associated with reduced risk of developing psychotic disorders in individuals with diabetes compared with standard diabetic treatments^206^. Moreover, structured exercise interventions have demonstrated robust effects on multiple hallmarks of aging^207^, and have been associated with improved cognitive function and reduced inflammatory markers in psychotic disorders^208^. Exercise, therefore, offers a low-cost, low-risk strategy to delay biological aging in this population.

Together, these novel aging-related targets broaden the scope of possible interventions and highlight the need for multimodal treatment strategies for psychotic disorders that are grounded in aging biology.

## Literature gaps and future work

Important knowledge gaps remain to advance our hypothesis of accelerated aging as a framework for multisystem psychotic disorders. There is a great need for longitudinal, multimodal studies that span brain and peripheral systems to map the temporal dynamics and interactions of organ-specific aging. Bringing in evidence from areas such as stress reactivity, a full review of the role of the autonomic nervous system, and sleep, which are outside the scope of our initial review, would widen our knowledge of pathophysiological mechanisms across central and peripheral systems and could offer entirely novel families of druggable targets for therapeutic interventions. All models require large harmonized data, and this may be particularly relevant in BrainAge, where imaging can be impacted by scanner and protocol variability. Harmonization across biomarker aquation and at scale is a crucial next step. A key question is whether biological aging progresses synchronously across systems, or whether certain domains (e.g., metabolic, cardiovascular, or neural) act as upstream drivers of multisystem decline. It is also unclear whether advanced aging is primed from developmental timepoints: Are aging phenotypes inconsistently progressive (an accelerating and decelerating age gap), or does it remain consistently advanced in individuals compared to controls from birth? In addition, sex differences are rarely accounted for. This is despite evidence that both aging trajectories^209,210^ and psychotic disorders outcomes differ by sex^211^ to potentially influence biological vulnerability and treatment response in a sexually dimorphic manner. Current biomarker datasets are also heavily skewed toward individuals of European ancestry, limiting the generalizability of findings and the development of precision tools across diverse populations. Models of accelerated aging in psychotic disorders must also better incorporate environmental, social, and developmental factors, including life history of trauma, deprivation, and early-life stress. These factors shape biology across the lifespan and may interact with aging pathways in modifiable ways to exacerbate an individual’s risk for psychotic disorders.

## Conclusion

In conclusion, a clearer understanding of aging processes in psychotic disorders could offer a novel path for both elucidating causal pathophysiology and translating insights into improved treatments. Current molecular and brain imaging signatures of aging are consistent with the view of psychosis as a multisystem disorder of accelerated biological aging. Future work should identify which aging mechanisms are most relevant to psychosis—particularly at illness onset—the interaction between aging systems, and where targeted interventions have the greatest impact signal for causality. Such knowledge could inform stratified prevention strategies and novel therapeutics aimed at reducing multimorbidity and premature mortality in this vulnerable population.

## Data Availability

No datasets were generated or analyzed during the current study.
